# Small Dimension–Big Impact! Nanoparticle-Enhanced Non-Invasive and Intravascular Molecular Imaging of Atherosclerosis In Vivo

**DOI:** 10.3390/molecules25051029

**Published:** 2020-02-25

**Authors:** Tobias Lenz, Philipp Nicol, Maria Isabel Castellanos, Leif-Christopher Engel, Anna Lena Lahmann, Christoph Alexiou, Michael Joner

**Affiliations:** 1 German Heart Centre Munich, Technical University of Munich, Lazarettstraße 36, 80636 Munich, Germany; tobiasbe.lenz@googlemail.com (T.L.); philipp.nicol@gmx.de (P.N.); mariacaste@gmail.com (M.I.C.); leifengel@hotmail.com (L.-C.E.); annalena@lahmann.at (A.L.L.); 2 DZHK (German Centre for Cardiovascular Research), partner site Munich Heart Alliance, 80802 Munich, Germany; 3 Department of Oto-rhino-laryngology, head and neck surgery, Section of Experimental Oncology and Nanomedicine (SEON), Else Kröner-Fresenius-Stiftung-Professorship, University Hospital Erlangen, 91054 Erlangen, Germany; c.alexiou@web.de

**Keywords:** molecular imaging, nanoparticles, atherosclerosis

## Abstract

Extensive translational research has provided considerable progress regarding the understanding of atherosclerosis pathophysiology over the last decades. In contrast, implementation of molecular in vivo imaging remains highly limited. In that context, nanoparticles represent a useful tool. Their variable shape and composition assure biocompatibility and stability within the environment of intended use, while the possibility of conjugating different ligands as well as contrast dyes enable targeting of moieties of interest on a molecular level and visualization throughout various imaging modalities. These characteristics have been exploited by a number of preclinical research approaches aimed at advancing understanding of vascular atherosclerotic disease, in order to improve identification of high-risk lesions prior to oftentimes fatal thromboembolic events. Furthermore, the combination of these targeted nanoparticles with therapeutic agents offers the potential of site-targeted drug delivery with minimized systemic secondary effects. This review gives an overview of different groups of targeted nanoparticles, designed for in vivo molecular imaging of atherosclerosis as well as an outlook on potential combined diagnostic and therapeutic applications.

## 1. Introduction

Cardiovascular atherosclerosis is considered a worldwide public health concern. Despite the emerging therapeutic advances, cardiovascular atherosclerosis remains a major cause of mortality and morbidity worldwide [1]. Cardiovascular atherosclerosis most often manifests as coronary artery disease (CAD) resulting in stable angina pectoris, acute coronary syndrome and sudden cardiac death; the second most frequent location of manifestation is cerebrovascular disease leading to transitory ischemic attack (TIA) and stroke; peripheral artery disease (PAD) represents another location of clinical manifestations leading to limb and visceral ischemia with rising prevalence over the last decades [2].

Acute coronary arterial thrombosis has been shown to arise from distinct morphological entities, which all result in thrombotic occlusion of the affected epicardial vessel leading to acute myocardial ischemia [3]. While plaque rupture has been identified as the most frequent underlying pathological substrate of coronary arterial thrombosis accounting for approximately two thirds of acute coronary events, plaque erosion has recently been recognized as a common cause of arterial thrombosis with rising prevalence especially in younger patients. Calcified nodule and acute plaque fissure are less frequent morphological correlates of acute coronary arterial thrombosis, where etiological factors and pathophysiology still remains unclear [4]. Survival of patients presenting with acute myocardial infarction has drastically improved since the introduction of percutaneous coronary intervention (PCI). In contrast, coronary angiography and PCI of angiographically severe stenosis in stable disease are still failing to prove equally capable of reducing mortality, since stenosis severity as assessed by angiography alone allows only very limited insight into underling pathophysiological processes and therefore does not reliably predict the individual risk for progression to future adverse events. Among the most identified risk factors of vulnerable plaque rupture are: increased lipid content (>40%), decreased collagen content with a thinned fibrous cap, and increased inflammatory cell infiltrate (abundant macrophages and to a lesser extent T- cell lymphocytes) [5]. Owing to the disparity of potentially disastrous consequences of acute cardiovascular and cerebrovascular events on the one hand, and the lack of established diagnostic tools to differentiate underlying pathologies of stable cardiovascular atherosclerotic disease on the other, the identification of such vulnerable plaques represents an important clinical need.

The currently available imaging modalities in clinical practice such as computed tomography (CT), magnetic resonance imaging (MRI), intravascular ultrasound (IVUS) and optical coherence tomography (IVOCT) are capable of delineating certain features of vascular anatomy. Nevertheless, these modalities do not routinely provide information regarding the underlying pathophysiological processes implicated in disease development and its complications. Whereas other disciplines can rely on biopsies when medical imaging reaches its limits, detailed assessment of pathophysiological processes of cardiovascular atherosclerotic disease at a biochemical, cellular or molecular level, relies on further refinement of the above-mentioned imaging techniques. Along these lines, molecular imaging offers both researchers and clinicians the chance to visualize anatomical and functional information within living cells, tissues and intact subjects [6]. The following review aims to provide a brief overview of basic principles and preclinical research approaches exploring different potential targets and specifically designed nanoparticles in the context of functional imaging of atherosclerosis, as well as an outlook on clinical applications. Considering the abundance of valuable preclinical research regarding this topic, focus was placed on studies that demonstrate proximity to clinical translation.

## 2. Basic Principles of Nanotechnology

Nanoparticles refer to particles that have one or more dimensions of 100 nm or less. Owing to the unique properties conferred by their size, functionalization abilities and modular structure, biomedical nanoparticles have continuously been exploited and used in the field of medical imaging. They serve as contrast agents for molecular imaging modalities and some are clinically employed as diagnostics as well as delivery vehicles for pharmacotherapeutics, consequently being referred to as “theranostics” [7].

One of the most frequently applied groups of nanoparticles for non-invasive atherosclerosis imaging are iron oxide-based nanoparticles (IONs). In simple terms, IONs are composed of a magnetite (Fe_3_O_4_) or maghemite (γ-Fe_2_O_3_) core and a coating ensuring hydrophilicity and biocompatibility, even for human application [8,9]. While their paramagnetic properties ensure suitability for magnetic resonance imaging, facilitated uptake by activated phagocytic cells of atherosclerotic plaques enables recognition of nanoparticles on a cellular level. Conjugation of different ligands allows targeted functional visualization of atherosclerosis features on a molecular level. Superparamagnetic iron oxide nanoparticles (SPIONs) are subdivided according to their size, core crystallinity and form of surface modification [10,11].

The second most common approach to formulate nanoparticles for molecular imaging uses polar lipids in micellar or liposomal arrangements. Less frequently, NPs are composed entirely of polymers or dendrimers, serving as both structure and coating in order to ensure biostability and biocompatibility. Surface modifications of such nanoparticles enable targeting of specific cell types, membrane proteins, enzymes or inflammatory mediators. Conjugation of peptide sequences or mimicking of whole endogenous particles and molecules are among the different approaches, used to achieve such selectivity for predefined targets. Unlike IONs, most other basic NP structures do not rely on inherent paramagnetic properties. Therefore, incorporation of paramagnetic contrast agents like gadolinium, radiopaque agents like gold, radiotracers, or fluorescent molecules like rhodamine, cyanine and quantum dots, ensures visualization throughout different imaging modalities [12].

## 3. Non-Invasive Nanoparticle-Enhanced Molecular Imaging of Atherosclerosis

Most non-invasive molecular imaging approaches follow the same basic principle, combining a conventional method of cross-sectional imaging with a contrast agent specifically targeting pathophysiological processes involved during progression of atherosclerosis. Potential targets of interest regarding molecular imaging of atherosclerosis are endothelial cells, macrophages and smooth muscle cells (SMC) as well as respective surface proteins, cell adhesion molecules, lipoproteins, enzymes and extracellular matrix. Due to their convertible shape and size as well as other favourable physiochemical properties, nanoparticles represent a dynamic enhancement to conventional contrast agents in the context of molecular imaging [12].

### 3.1. Endothelial Dysfunction

Endothelial cells have been the focus of molecular imaging within recent years owing to their critical involvement in initiation of atherosclerosis; in particular, endothelial cell adhesion molecules have been identified as targets of interest for molecular imaging approaches. Among the best studied of those adhesion molecules is VCAM-1, a surface protein, expressed predominantly on activated endothelial cells, promoting transmigration of inflammatory cells into the newly developing atherosclerotic plaque. In a landmark study in the field of multimodal molecular imaging using iron oxide-based nanoparticles, Jaffer et al. developed a monocrystalline iron oxide magnetic nanoparticle (MION) covered with dextran, cross-linked (CLIO) and aminated, resulting in a magnetic nanoparticle (MNP) with an overall size of 38 nm and an average of 62 primary amines available for conjugation. Reaction with fluorochrome cyanine 5.5 then yielded the final near infrared fluorescent (NIRF) magneto-fluorescent nanoparticle (MFNP) [13]. On this basis, Nahrendorf and colleagues further developed a magneto-fluorescent nanoparticle coated with a previously identified VCAM-1-targeting peptide sequence (VINP-28). Application of this novel imaging agent in ApoE-deficient (ApoE^−/−^) mice showed strong accumulation in the endothelial layer as well as subendothelial regions of mouse atherosclerotic lesions as assessed by in vivo MRI-signal attenuation 48 h after injection, and disclosed by ex vivo immunohistochemistry. Furthermore, VINP-28-MNP was able to distinguish different degrees of VCAM-1 expression between atorvastatin-treated mice and control as well as early atherosclerotic changes in juvenile animals. Furthermore, VINP-28-incubation of a freshly resected human carotid endarterectomy specimen consistently showed MRI-signal alteration and plaque enhancement by fluorescence imaging as well as colocalization with VCAM-1-expressing cells [14]. 

The majority of nanoparticles are spherical. In contrast, Bruckman and colleagues modified the plant virus tobacco mosaic virus (TMV), in an interesting approach generating a tubular VCAM-1-targeting NP. For this purpose, interior and exterior amino acids of TMV’s coat protein were used to introduce contrast agents and peptide ligands, coupled through alkyne ligation handles. The VCAM-TMV created in this manner is coated with a VCAM-1 specific peptide, as well as PEG, while the interior is modified with gadolinium and Cy5 fluorescent dye. Application of VCAM-TMV in the established model of ApoE-deficient cholesterol-fed mice showed considerably increased signal-to-noise ratio in MRI at 90 min after injection, as compared to free gadolinium and phosphate-buffered saline (PBS). Ex vivo fluorescence imaging and immunohistology confirmed VCAM-TMV accumulation at the intima–media interface of 70% of atherosclerotic plaque sections, compared to no accumulation in healthy tissue, as well as no accumulation of control NPs in the atherosclerotic plaque [15].

Molecular imaging of activated and inflamed endothelium via VCAM-1 targeting agents provides the potential to visualize an early and still reversible stage of atherosclerosis development. From a translational perspective, successful ex vivo application of VINP-29-MNFP in human autopsy specimens, as well as a SNR-peak at only 90 min after injection of VCAM-TMV, appear promising.

### 3.2. Macrophages

Macrophages mark the transition from pathological intimal thickening and intimal xanthoma to more advanced atherosclerosis. One of the pioneering NPs used for macrophage imaging is the dextran-coated, cross-linked and fluorescent magnetic nanoparticle developed by Jaffer et al., introduced above. Quantification of uptake of these MFNPs by different murine cell-types via flow cytometry showed the greatest uptake by activated macrophages, followed by smooth muscle and endothelial cells. In vivo magnetic resonance imaging of ApoE-deficient mice showed considerable nanoparticle uptake in aortic atheroma after injection of MFNPs, as confirmed by ex vivo fluorescence reflectance imaging, fluorescence microscopy and histopathology. Consistently, in vivo uptake of MFNP was higher in macrophages than in smooth muscle and endothelial cells [13]. Numerous further studies have verified the suitability of iron oxide-based nanoparticles for molecular imaging of atherosclerosis, particularly targeting macrophages [16].

As substantial macrophage infiltration into early atherosclerotic lesions is considered a key feature in the progression from stable to vulnerable plaque, multimodality molecular imaging approaches strongly target their reliable detection. In that sense, Nahrendorf and colleagues enhanced a dextran-coated, cross-linked and, for additional conjugation, aminated iron oxide nanoparticle with a near-infrared fluorochrome and the radiotracer ^64^Cu, derivatized with the chelator DTPA. This combined paramagnetic, positron-emitting and fluorescent targeted iron oxide nanoparticle (^64^Cu-TNP) was then tested against ^18^FDG in an ApoE-deficient mouse model. CT-contrast was achieved by additional infusion of an iodinated contrast agent. PET-CT imaging 24 h after application of ^64^Cu-TNP showed accumulation in atherosclerotic arteries, as corroborated by in vivo MRI and confirmed by ex vivo histology and macroscopic fluorescence reflectance imaging. Conjunction of flow cytometry of digested aortas and fluorescence microscopy revealed uptake of ^64^Cu-TNP predominantly by macrophages, but also other inflammatory cells, as well as activated endothelial and smooth muscle cells. Interestingly, these cell types did not show NP-uptake in nonatherosclerotic wall sections. Comparison to ^18^FDG administered one hour before imaging revealed slightly higher and longer persistent signal enhancement for ^64^Cu-TNP [17].

In addition to detection of macrophages through simple phagocytosis of the imaging agent, visualization of the characteristic infiltration of the vascular wall by these cells can be achieved by targeting of specific epitopes. An interesting example in this sense is a study by Tarin et al. The group crafted a nanoparticle, consisting of a gold-coated iron oxide core, covered with mannose and carboxylic acid attached through thiol ligands. Exploiting the high affinity of protein G to the FC region of antibodies, covalent binding of this immunoglobin-binding protein to carboxylic moieties then allowed grafting of CD163-specific monoclonal antibodies to the NP [18]. CD163 is a scavenger receptor with high affinity to the haemoglobin–haptoglobin complex. It has been linked to promoted angiogenesis, vascular permeability and inflammation following intraplaque haemorrhage, thereby adding to the formation and destabilization of advanced atherosclerotic plaque [19]. Injection of the CD163-targeting gold NPs and a non-targeted control NP to ApoE-deficient as well as wild type mice revealed significant MRI-signal decrease in the abdominal aortic wall at 48 h post-injection of the targeted NP in ApoE-deficient mice. Neither control NPs nor wild type mice showed considerable signal variation. Immunohistochemical validation confirmed atherosclerotic lesions in the MRI-scanned regions [18].

Another remarkable proof-of-principle study exploited the natural affinity of high-density lipoproteins (HDL) to macrophages in atherosclerotic plaques. Composed of a phospholipid monolayer with intercalated apolipoproteins, encapsulating triglycerides and cholesterol esters inside a hydrophobic core and, given their dimension of less than 20 nm, HDL particles have strong resemblance with synthetic lipid-based nanoparticles. This implies that minor adjustments like incorporation of a contrast agent would yield a nanoparticle fit to be used for molecular imaging, while preserving the characteristics of physiological HDL. Cormode et al. designed an HDL-like nanoparticle with a phospholipid monolayer containing ordinary phospholipids as well as optional gadolinium- or rhodamine-labeled phospholipids and additional incorporation of lipoprotein A1. The multimodality NP was completed by inclusion of either one of three different inorganic nanocrystals with varying imaging properties into the hydrophobic core: x-ray attenuating gold (AU-HDL), paramagnetic iron oxide (IO-HDL) or fluorescent quantum dots (QD-HDL). Respective control NPs incorporated the same nanocrystals but lacked HDL-like structure. After in vitro experiments confirmed high affinity to macrophages as well as suitability for CT, MRI and fluorescence imaging, in vivo imaging efficacy was assessed, injecting the three different HDL-like NPs and respective control particles into ApoE-KO mice after a 10-month high-cholesterol diet. Small animal MRI revealed a clear increase in signal intensity in the aortic wall for AU-HDL and QD-HDL, both containing gadolinium-conjugated phospholipids, and a clear decrease for FeO-HDL. The corresponding control particles showed no significant difference as compared to the pre-contrast situation. Confocal microscopy and fluorescence imaging reassured colocalization of nanoparticles and macrophages inside the atherosclerotic aortic wall. Clearly delineated bright areas in ex vivo CT imaging of an aorta specimen taken from mice injected with AU-HDL compared to control NP indicated applicability of nanocrystal core HDL-like NPs for CT-imaging [20].

The abundance of nanoparticles aiming at macrophages in atherosclerotic plaques, exemplifies the importance of these cells in atherosclerosis development. Various modalities have proven feasible to non-invasively assess disease burden in atherosclerotic animal models targeting macrophages. In addition, the study by Tarin et al. provides a way to selectively image specific subtypes of the monocytic lineage in order to distinguish different stages of plaque progression.

### 3.3. Vascular Smooth Muscle Cells

In addition to endothelial cells and macrophages, another cell type critically involved in all stages of atherosclerosis development is the vascular smooth muscle cell (VSMC). These cells are able to differentiate into various phenotypes, contributing to arterial homeostasis as contractile, synthetic or phagocytic cells. However, in conjunction with detrimental cardiovascular risk factors like diabetes mellitus, hyperlipidaemia or hypertension disturbing this balance, VSMCs also play a considerable role in the initiation and progression of atherosclerotic changes by facilitating the retention of lipoproteins inside the vessel wall through production of negatively charged extracellular matrix (ECM) components, maintaining an inflammatory milieu through cytokine secretion and recruitment of macrophages as well as transformation into VSMC-derived foam cells [21]. Profilin-1 is an intracellular actin-binding protein involved in cytoskeletal dynamics that has also been observed to be highly expressed in human atherosclerosis and to exert direct atherogenic effects on VSCMs [22]. Zhang and colleagues made use of this fact when they designed a polymer-coated iron oxide-based MNP, conjugated to a profilin-1 antibody, Cy5.5 and cyclodextrin (CD), and established a nanocarrier for hydrophobic drugs (PFN1-CD-MNP). In vitro testing confirmed upregulated PFN1-expression in immortalized murine aortic smooth muscle cells treated with oxidized LDL (oxLDL), as well as high affinity of PFN1-CD-MNP to these cells. The quality of PFN1-CD-MNP as a diagnostic agent imaging atherosclerosis was tested using the ApoE-deficient atherosclerotic mouse model. MRI before and 24 h after administration of PFN1-CD-MNP showed a significant T2-weighted signal attenuation in the carotid artery wall of ApoE^−/−^ mice as opposed to a lower attenuation after administration of the untargeted control NP (CD-MNP). Ex vivo fluorescence imaging and histological assessment confirmed the promoted deposition of the targeted NP in atherosclerotic plaques compared to the wild type control group. Furthermore, PFN1-CD-MNPs were loaded with rapamycin via cyclodextrin (Rap@PFN1-CD-MNP) and administered every three days intraperitoneally to ApoE-deficient mice after two months of a high-fat diet for a time period of another two months. Compared to a saline control group, the untargeted therapeutic nanoparticle, Rap@PFN1-CD-MNP, seemed to reduce plaque burden as assessed by histology and ex vivo fluorescence imaging, combined with less toxicity as indicated by blood biochemistry analysis [23].

Although the authors of this study failed to provide detailed statistics, such as exact p-values, as well as information on the number of animals in the respective experimental groups and the diagnostic accuracy of the targeted NP, which was not tested against a control group of healthy animals, the approach adds not only another molecular target but also another cell type to the repertoire of targeted nanoparticles applied for molecular imaging of atherosclerosis. In addition, it serves as a good example for the theranostic potential of this class of nanomaterials.

### 3.4. Neovascularization

Another important factor of atherosclerosis progression is neovascularization. Starting in early atherogenesis, when neointimal thickening prompts hypoxia leading to sprouting of new capillaries with increased permeability for lipoproteins and leukocytes, facilitated lipid deposition and inflammatory cell recruitment cause progression of early atherosclerotic lesions into more advanced plaques. Extravasation of red blood cells (RBCs) causing intraplaque hemorrhage increases plaque burden and vulnerability [24,25]. Research regarding angiogenesis in tumor growth and metastasis identified increased expression of endothelial integrins in nascent neovessels. One of the best known representatives is αvβ3-integrin [26]. Winter and colleagues formulated a nanoparticle using perfluorocarbon encapsulated by a lipid-monolayer in a micellar fashion, bearing gadolinium and a peptidomimetic RGD-sequence in order to bind αvβ3-integrin. In a preclinical study using atherosclerotic New Zealand white rabbits, MRI showed significantly increased signal enhancement 120 min after treatment with targeted NPs vs. control. In cholesterol-fed rabbits, histology and immunostaining revealed intimal thickening with concomitant expansion of PECAM-positive vasa vasorum. Colocalization of PECAM and αvβ3 near the adventitia–media interface indicated a subpopulation of angiogenic vessels in hyperlipidaemic animals [27]. This study illustrates the potential of nanoparticle-enhanced molecular imaging to detect the expansion of vasa vasorum in early atherosclerosis. 

### 3.5. Inflammation

A substantial part of inflammatory cell transmigration into atherosclerotic plaque via VCAM-1 on activated endothelial cells is mediated through integrin α4β1, an integrin expressed on different types of circulating leukocytes. In an interesting approach targeting the cell-mediated inflammatory part of atherosclerosis development, Woodside at al. developed a liposomal nanoparticle composed of different polar lipids bearing gadolinium, polyethylene glycol (PEG), a specifically developed selective integrin α4β1 antagonist (THI0567), and rhodamine, a fluorescent dye. These nanoparticles, along with a non-targeted liposomal-Gd agent serving as control, were then injected intravenously into ApoE-deficient mice that had previously been on a 10-week high-cholesterol diet. In vivo MRI showed significantly higher aortic wall signal enhancement after administration of the THI0567-targeted liposomal-Gd agent as compared to the control agent. Ex vivo histology and confocal fluorescence imaging revealed accumulation of the targeted liposomes in oil-red positive atherosclerotic plaque and colocalization with cells of the monocytic lineage [28].

Following pathological intimal thickening, endothelial dysfunction, accumulation of extracellular lipid and inflammatory cell migration into the early atherosclerotic lesion, progression to advanced and vulnerable atherosclerotic plaque is characterized by a growing acellular portion of the plaque eventually leading to the formation of the so-called necrotic core. Besides apoptosis of SMCs and macrophages, decreased expression of extracellular proteoglycans are considered causal in this context. Activated matrix metalloproteinases (MMPs), synthesized by macrophage- and SMC-derived foam cells, are believed to be responsible for this degradation of extracellular matrix proteins causing progression of early to late fibroatheroma, as well as vulnerable plaque [29]. MMP expression is regulated by an extracellular receptor expressed by various cell types, including macrophages, SMCs and endothelial cells, called extracellular matrix metalloproteases inducer (EMMPRIN). Ramirez-Carracedo et al. used a paramagnetic micellar nanoparticle composed of a monolayer of polar lipids conjugated with gadolinium, rhodamine, PEG and AP9, an EMMPRIN-specific binding peptide (NAP9). In order to assess the ability of NAP9 to non-invasively visualize atherosclerotic lesions by MRI, NAP9 or control NP were intravenously injected into ApoE/ NOS3-deficient, as well as single ApoE-deficient, mice on a high-cholesterol diet. In vivo MRI sequences showed signal enhancement in the carotid wall after NAP9-injection as compared to the control NP. Carotid sections further revealed a strong colocalization of EMMRPIN and NAP9 in atherosclerotic plaques, confirming the potential of NAP9 for molecular imaging targeting inflammation promoting atherosclerotic plaque progression [30].

In addition to the wide range of nanoparticles, designed to visualize macrophage burden, targeting of integrin α4β1-expressing cells allows imaging of overall inflammatory cell burden. One step further towards vulnerable plaque, the approach by Ramirez-Carracedo et al. aims at inflammatory activity of the cells in question.

### 3.6. Lipids

Oxidized LDL-receptor-1 (LOX-1) is known as the major receptor for oxidized LDL (oxLDL), expressed chiefly on endothelial cells, but also smooth muscle cells and cells of the monocytic lineage. LOX-1 has been linked to an abundance of atherosclerosis-promoting factors and is believed to play a key role in both early atherosclerosis development as well as progression. This assumption rests on evidence for activation and upregulation of LOX-1 by hypertension, hyperglycemia, inflammatory stimuli and oxidized lipoproteins, and subsequent induction of endothelial dysfunction, inflammation, foam cell formation, apoptosis and platelet aggregation [31]. These diverse interactions have brought LOX-1 to the attention of researchers dedicated to molecular atherosclerosis imaging. Dayuan Li et al. developed a liposomal nanoparticle consisting of polar lipids, cholesterol and PEG-derivates and a cyanine-based fluorescent dye, coupled with an anti-LOX-1-antibody as well as lipids containing either gadolinium for MRI or indium 111 for SPECT-imaging. In addition to the well-established ApoE^−/−^ mouse model, LDLR^−/−^ and LDLR^-/-^LOX-1^−/−^ mouse models were used to test properties and suitability of these NPs for visualization of LOX-1-expression. Immunohistology and fluorescence imaging showed high LOX-1 expression in atherosclerotic plaques as well as colocalization mainly to macrophages, apoptotic cells and MMP-9. In vivo SPECT and phosphor imaging of ApoE-deficient mice showed hot spots in the atherosclerotic regions of the aortic arc. MRI in LDLR-deficient animals revealed strong gadolinium enhancement in the aortic root and arch after injection of the LOX-1-NP. Size and localization of atherosclerotic plaques were confirmed by ex vivo fluorescence imaging and H&E staining. LRLR and LOX-1 double-knockout mice showed fewer atherosclerosis and neither LOX-1-NP binding nor GD-enhancement [32]. 

In a similar context, Wen and colleagues used an ApoE^-/-^ mouse model of high fat diet and perivascular collar induced atherosclerosis to specifically visualize oxidized LDL-loaded foam cells. For that purpose, ultrasmall superparamagnetic iron oxide nanoparticles (USPIOs) were PEG-coated and conjugated to anti-mouse oxLDL-antibodies. Preliminary in vitro assessment showed strong macrophage uptake after pre-exposition of both NPs and macrophages to oxLDL. Negligible NP-uptake by raw macrophages without oxLDL pre-exposition of either macrophages or NPs shows the ability of the targeted NPs to visualize oxLDL-loaded foam cells in a hypercholesteremic environment. In vivo MRI 8 and 24 h after injection of targeted NPs confirmed significant signal loss in atherosclerotic carotid arteries of ApoE^−/−^ animals. Furthermore, histology confirmed NP-deposition in oxLDL-enriched macrophages [33].

Another multimodality approach aiming at oxidized LDL was proposed by Pellico at al., based on pretargeted bioorthogonal molecular imaging. This imaging technique requires a biomolecule targeting the epitope of interest and an imaging agent, both labeled with complementary chemical compounds to secure selective biorthogonal conjugation. The method of choice for this particular study is bioorthogonal tetrazine ligation, a rapid reaction between a tetrazine derivate (TZ) and trans-cyclooctene (TCO). Pellico and colleagues used a citrate-coated small iron oxide nanoparticle combined with a ^68^Gadolinium isotope to generate a nano-radiomaterial suitable for PET- and MR-imaging (^68^Ga-NRM), conjugated to a tetrazine derivate (^68^Ga-NRM-TZ) through amide formation. In order to direct the imaging agent to the epitope of interest, E-06, a mouse monoclonal IgM antibody (Ab) targeting oxidized LDL, oxidized HDL and other proteins covalently modified by oxidized phospholipids, was conjugated to trans-cyclooctene (Ab-TCO). In vivo application was carried out using the established ApoE^−/−^ atherosclerotic mouse model after in vitro verification of the bioorthogonal reaction capability of both antibody and NRM and confirmation of specificity of the antibody, radioactive labeling stability, radiochemical purity as well as conservation of positive signals for PET and MRI of the NRM. The full pretargeted approach, meaning the injection of Ab-TCO followed 24h later by injection of ^68^Ga-NRM-TZ to ApoE^-/-^mice after 25 weeks of high fat diet and in vivo PET scans another hour after NRM-injection, showed clear accumulation of signal in several consecutive planes of the aortic arc. Specific accumulation due to the biorthogonal reaction and selective recognition of oxidized phospholipids was confirmed by a control group of C57BL/6 mice and ApoE^−/−^ mice on a normal diet, as well as control particles (Ga-NRM without tetrazine) and a blocking experiment with the oxLDL antibody injected prior to the full experiment in order to block most of the recognition sites. Ex vivo MRI of the aorta specimen of ApoE^-/-^ mice injected with Ga-NRM and Ga-NRM-TZ allowed researchers not involved in the previous experiments to distinguish between control and experimental specimens, due to hyperintense areas representing atherosclerotic lesion sites [34].

Visualization of “foamy macrophages”, oxidized LDL and LOX-1, an important junction of various atherosclerosis-specific pathological pathways, gives credit to the role of oxidized lipoproteins in atherosclerosis development and offers considerable potential to target a pathognomonic macrophage subtype.

### 3.7. Apoptosis and Advanced Disease

Apoptosis of macrophages and foam cells mark a “point of no return” in the stepwise progression from pathologic intimal thickening towards advanced and increasingly vulnerable atherosclerotic lesions with necrotic core [35]. Phosphatidylserine (PS) is a phospholipid, localized in the inner layer of healthy cell membranes. In apoptotic cells, PS is no longer restricted to the cytoplasmic membrane leaflet, but exposed on the outside of activated and dying cells. In order to visualize the transition to advanced and vulnerable atherosclerotic plaque, van Tilborg et al developed a micellar nanoparticle, by assembling different polar lipids bearing the fluorescent dyes Cy5.5 and rhodamine, the MRI-contrast agent gadolinium, and different PEG-based polymers, covalently coupled to annexin A5, a known phosphatidylserine ligand. After in vitro verification of the selective PS-binding capacity of the annexin A5 micellar NPs (A5-mNPs) using ellipsometry and apoptotic Jurkat cells, A5-mNPs were tested in vivo in ApoE-deficient cholesterol-fed mice. MRI at 24 h after injection of A5-mNP showed significant increase in signal intensity by 10.7 ± 1.7% within the atherosclerotic vascular aortic wall, compared to 6.7 ± 3.4% after injection of control-NPs. Ex vivo near-infrared fluorescence and confocal fluorescence microscopy revealed multiple fluorescent hotspots at atherosclerosis predilection sites, as well as clear colocalization with macrophages and apoptotic cells in ApoE-deficient mice after administration of A5-mNP [36].

A marker that also seems to be connected to an advanced plaque pathology, without being clearly connected to a circumscribable cell type or stage of disease progression, is natriuretic peptide clearance receptor, also called natriuretic peptide receptor C (NPR-C). NPR-C is a cell surface protein involved in vascular homeostasis and remodeling, linked to features of advanced atherosclerotic disease and vulnerable plaque. Assessment of NPR-C expression in human carotid endarterectomy specimens (CEA) showed promoted expression in the superficial and deep intima of advanced carotid artery plaques as compared to minimally diseased specimens [37]. Based on these observations, Liu et al. proposed a polymeric nanoparticle targeting NPR-C. The NP consists of several methacrylate-based functional monomers, including ones conjugated to NPR-C ligand c-atrial natriuretic factor (CANF), as well as a DOTA chelator, assembled through controlled radical polymerization (CANF-comb). The final targeted nanoparticle carries ∼35 copies of the CANF peptide on the surface, as well as ∼105 copies of the DOTA chelate in the core for ^64^Cu labeling, enabling PET-imaging (^64^Cu-CANF-comb). A rabbit double-injury atherosclerosis model, involving a high fat diet followed by air-desiccation, inducing injury of one of the femoral arteries and subsequent balloon overstretching of the same segment 4–6 weeks later, was used to assess the potential of ^64^Cu-CANF-comb to target and visualize NPR-C expression in vivo using PET-imaging. PET at different timepoints (TP), starting with the first injury by air-desiccation (TP1), and followed by two different time points after balloon angioplasty (TP2 and TP3), revealed a 2.5-fold higher standardized uptake value (SUV) as compared to the sham-operated side at TP1 and gradually decreasing but of the same magnitude at TP2 and TP3. Furthermore, targeting specificity of ^64^Cu-CANF-comb was confirmed through much higher accumulation as compared to non-targeted ^64^Cu-comb, as well as a more than 50% decrease after administration of an excess amount of non-radiolabeled CANF-comb serving as the competitive ligand. Histopathological characterization of a representative rabbit artery specimen showed primary neointima containing increased numbers of foam cells and smooth muscle cells at the injury site, whereas immunofluorescent staining showed high NPR-C expression in the neointimal layer of the plaque. Interestingly, immunohistopathological characterization of an atherosclerotic human CEA specimen showed a comparable NPR-C expression in the deep intimal layer, and autoradiography using ^64^Cu-CANF-comb confirmed a significant tracer binding to NPR-C positive areas, sustaining translational potential [38].

While the majority of the aforementioned approaches started at earlier stages of atherosclerosis pathogenesis, visualization of apoptotic cells as well as targeting natriuretic peptide receptor-C aims at advanced fibroatheroma and thus consequently vulnerable atherosclerotic plaque.

### 3.8. Thrombosis

If primary prevention fails or if acute coronary syndrome and stroke are first manifestations of atherosclerotic disease, vulnerable atherosclerotic plaques cause arterial thrombosis. In the case of occlusive thrombosis, clinical presentation is in most cases pathognomonic and urgent angiography and revascularization are the next diagnostic and therapeutic steps. On the other hand, clinically silent thrombi are known to contribute to atherosclerosis progression and formation of luminal narrowing, and therefore represent an interesting target in the context of molecular imaging [35]. In order to selectively image thrombosis, Kwon et al. developed a gold nanoparticle (AuNP) coated with silicon dioxide linked to a thrombin-activatable fluorescent peptide (TAP). While the AuNP provides X-ray absorption and therefore serves a CT contrast dye, the thrombin-activatable fluorescent peptide, consisting of a thrombin-specific cleavable peptide linked to Cy5.5, enables near infrared fluorescence imaging. Preliminary in vitro testing of the final NP (TAP-SiO_2_@AuNP) with micro-CT phantom images revealed a dose-dependent contrast effect. Furthermore, the NIRF signal appeared to be quenched when imaging the untreated NP and significantly increased after incubation with thrombin. In vivo evaluation of TAP-SiO_2_@AuNP was performed in an established mouse model of in-situ arterial thrombosis, induced by a FeCl_3_-soaked filter paper placed on the exposed left distal common carotid artery (CCA). Starting 30 min after thrombus induction and another 20 min after venous injection of TAP-SiO_2_@AuNP, strong NIRF signals could be observed at the site of CCA thrombus for up to two hours. Micro-CT imaging showed accumulation and subsequent X-ray attenuation of TAP-SiO_2_@AuNP at the site of thrombosis. Finally, simultaneous NIRF-imaging and micro-CT showed NIRF-signal indicating high thrombin activity colocalized to relative brightness in the thrombotic lesion generated by radiopaque AuNP. Histology confirmed accumulation of TAP-SiO_2_@AuNP within the thrombus, most likely due to its size-dependent capturing property [39].

Using a similar animal model of FeCl_3_-induced thrombosis in the abdominal aorta of rats, Zhong et al. tested a NP based on paramagnetic iron oxide and Indian ink, two established contrast agents for MRI and photoacoustic imaging respectively, integrated in a polymer structure. Furthermore, NPs were linked to cRGD- as well as GA-EWVDV-ligands in order to specifically target glycoprotein IIb/IIIa and P-selectin on activated platelets. In vivo photoacoustic imaging 30 min after administration of the thus designed dual-targeted dual-modality NP (DDNP) showed increased signal intensity at the site of wall-adherent thrombus, as confirmed by conventional ultrasound. MRI-signal area at the site of induced thrombus decreased significantly as compared to non-targeted control NPs. Subsequent histopathological assessment confirmed localization of DDNPs on thrombus material [40].

In addition to the above-mentioned considerations regarding clinically silent thrombi, approximately one third of acute coronary syndromes are believed to be caused by thrombosis on the ground of plaque erosion [41]. Considering that optimal treatment of this entity is a matter of ongoing debate and increasing focus is placed on conservative approaches, non-invasive imaging of thrombus could offer both diagnostic potential as well as means to follow up on antithrombotic treatment effects.

## 4. Invasive Nanoparticle-Enhanced Molecular Imaging of Atherosclerosis

In contrast to the large number of non-invasive approaches of nanoparticle-enhanced molecular imaging of atherosclerosis, their invasive counterparts are limited. So far, the lion’s share of molecular imaging approaches in this field have focused on combinations of optical coherence tomography (OCT) and intravascular ultrasound (IVUS) with near infrared spectroscopy (NIRS) or fluorescence (NIRF) [42]. In particular, NIRF has been applied in promising concepts of targeted molecular imaging. Specifically designed fluorescent contrast agents in combination with IVUS or OCT for anatomical imaging proved the ability to visualize fibrin deposition [43,44], endothelial injury [45] or inflammation [46].

Although a considerable number of fluorescent nanoparticles has been designed for non-invasive applications, only few studies have assessed their applicability for in vivo intravascular imaging so far. However, a remarkable study on this subject by Stein-Merlob et al. showed that fluorescent nanoparticles engineered for non-invasive imaging can successfully be used as a contrast agent for intravascular near infrared fluorescence. The group used CLIO-Cyam7, a crosslinked iron oxide, derivatised with the near infrared cyanine fluorophore Cyam7 in a rabbit model of atherothrombosis. New Zealand white rabbits were fed a high cholesterol diet, before and after abdominal aortic balloon injury, in order to induce atheroma development. Thrombosis was triggered by injection of Russel’s viper venom and histamine in a predefined order. Visualization of fluorescent NPs was achieved by a custom-built 2D intravascular NIRF imaging catheter, while two-stage anatomical assessment was ensured by intravascular ultrasound. In vivo intravascular NIRF before thrombosis triggering showed demarcated near infrared fluorescent signal in areas of IVUS-confirmed atheroma. Ex vivo fluorescence reflectance imaging still detected CLIO-Cyam7 in areas of atheroma 72 h post-injection. Subsequent fluorescence microscopy revealed NP-accumulation mainly in the superficial intima of atherosclerotic lesions in comparison to negligible NP-accumulation in healthy tissue. Histopathological assessment revealed CLIO-Cyam7-accumulation not only within macrophages, but also atheroma-associated endothelial and smooth muscle cells. Immunohistochemical as well as fluorescence stains corroborated the restriction of CLIO-Cyam7 to the superficial plaque surface in approximately two thirds of the animals. The remaining third showed evidence of accumulation near the intima–media boarder, correlating with induced neovascularization as shown by CD31-immunostaining. The hypothesis that accumulation of CLIO-Cyam7 exposes areas of impaired endothelial barrier function of both native arteries and newly developing neovessels was further strengthened by good correlation between penetration depth of the nanoparticles and the concomitantly injected Evans Blue. Lastly, atherothrombosis induction caused formation of adherent thrombus to 21 of 51 atheroma segments compiled from all rabbits. Quantitative analysis revealed significantly higher CLIO-Cyam7-exibition in these “thrombogenic” atheroma than in those that did not cause thrombus formation after triggering [47]. In addition to the successful proof-of-principle from the imaging standpoint, this study adds to the mechanistic understanding of iron-oxide nanoparticle accumulation in atheroma though disrupted endothelial integrity. Furthermore, increased endothelial permeability seems to favour atherothrombotic susceptibility, and fluorescent iron-oxide nanoparticles in combination with intravascular NIRF-imaging appear to offer the potential of identifying these “vulnerable plaques” in vivo.

Another intravascular molecular imaging approach that yielded preclinical in vivo application in a rabbit model of high cholesterol diet and balloon denudation similar to the one described above used ICAM-1-targeting echogenic liposomes in combination with intravascular ultrasound. Similar to VCAM-1, ICAM-1 is an adhesion molecule expressed on activated endothelial cells in early atherogenesis [48]. Kee et al. used an echogenic liposome consisting of various polar lipids, including one containing rhodamine that gains echogenicity through encapsulation of air (ELIP). Mouse monoclonal anti rabbit-ICAM-1 antibodies were conjugated to the echogenic liposomes through a thioether linkage (anti-ICAM-1 ELIP). Furthermore, nitric oxide (NO) containing ELIPs were prepared according to the same protocol but additional NO-encapsulation (NO-ELIP) was achieved. Intravascular anatomic and functional imaging was performed with a commercially available IVUS imaging catheter. While mere application of anti-ICAM-1 ELIP and subsequent intravascular ultrasound caused little signal change in the suspected areas of atheroma, application of NO-ELIP in combination with ultrasound activation demonstrated visually increased echogenicity, most probably due to increased vasodilation and permeability for targeted liposomes. Immunofluorescence microscopy and immunohistochemical staining of ICAM-1 confirmed pathological intimal thickening with high ICAM-1-expression as well as anti-ICAM-1 ELIP accumulation throughout all layers of the developing plaque after NO-pretreatment and ultrasound activation [49]. Although less closer to clinical translation than the preceding approach, the work of Kee and colleagues provides an additional path for the invasive molecular imaging of early atherosclerosis.

A third promising modality is intravascular photoacoustic imaging (IVPA). Photoacoustic imaging is a technique that provides information about tissue components derived by differences in optical absorption combined with photoacoustic feedback and has already been applied in vivo in combination with intravascular ultrasound in the preclinical setting [50,51]. However, targeted nanoparticle-enhanced approaches are currently still limited to an early stage of development. Wang et al. showed that plasmonic PEG-coated gold nanoparticles (Au-NP) internalized by murine macrophages exhibit an increased and broadened wavelength extinction spectrum as compared to the nanoparticles in solution, due to the plasmon coupling effect. Au-NPs in solution as well as NP-loaded macrophages were loaded into separate chambers of a rotating vessel-mimicking phantom. Subsequent imaging with a combined IVUS/IVPA-catheter prototype revealed photoacoustic signal of both NPs and loaded macrophages at the extension peak of the solved NPs (530 nm), whereas at an increased wavelength of 680 nm only the compartment filled with NP-loaded macrophages produced photoacoustic signal. Further refinement of the intravascular catheter-prototype and in vivo experiments need to follow in order to establish photoacoustic imaging as another feasible technique to visualize targeted nanoparticles in the context of atherosclerosis [52].

## 5. Conclusions and Theranostic Perspectives

**Figure 1:**
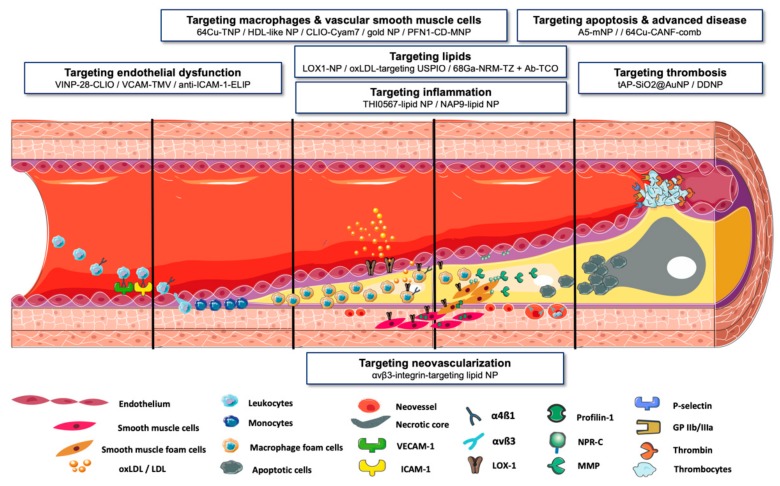
**Take-home figure. Molecular targets on different stages of atherogenesis: (VINP-28-MNP)** VCAM-1-targeting magneto-fluorescent NP [14] **(VCAM-TMV)** VCAM-1-targeting modified tobacco mosaic virus [15] **(anti-ICAM-1-ELIP)** ICAM-1-targeting echogenic liposomes [49] **(^64^CU-TNP)** paramagnetic, positron-emitting, fluorescent targeted iron oxide NP [17] **(HDL-like NP)** HDL-like multimodality NP [20] **(CLIO-Cyam7)** crosslinked iron oxide, derivatised with Cyam7 for near infrared fluorescence imaging [47] **(gold NP)** CD163-targeting gold NP [18] **(PFN1-CD-MNP)** profilin-1-targeting magneto-fluorescent nanoparticle [23] **(αvβ3-integrin-targeting lipid NP)** micellar nanoparticle bearing gadolinium and a peptidomimetic RGD-sequence binding αvβ3-integrin [27] **(LOX1-NP)** liposomal NP coupled with an anti-LOX-1-antibody, containing a fluorescent dye and either gadolinium or indium 111 [32] **(oxLDL-targeting USPIO)** ultrasmall superparamagnetic iron oxide NP conjugated to anti-mouse oxLDL-antibodies [33] **(^68^Ga-NRM-TZ + Ab-TCO)** paramagnetic, positron-emitting nano-radiomaterial connected via tetrazine ligation to antibody against oxidized phospholipids [34] **(THI0567-lipid NP)** liposomal NP bearing gadolinium, selective integrin α4β1 antagonist THI0567 and rhodamine [28] **(NAP9-lipid NP)** paramagnetic micellar NP conjugated with gadolinium, rhodamine and EMMPRIN-specific binding peptide NAP9 [30] **(A5-mNP)** micellar NP bearing Cy5.5 and rhodamine, gadolinium and polymers, covalently coupled to phosphatidylserine ligand annexin A5 [36] **(^64^Cu-CANF-comb)** NPR-C-targeting polymeric positron-emitting nanoparticle **(TAP-SiO2@AuNP)** gold NP coated with silicon dioxide linked to a thrombin-activatable fluorescent peptide [39] **(DDNP)** NP based on paramagnetic iron oxide and Indian ink linked to cRGD- and GA-EWVDV-ligands targeting glycoprotein IIb/IIIa and P-selectin on activated platelets [40].

Numerous nanoparticles targeting different stages of atherosclerosis development have demonstrated the potential of clinical translation through successful application in in vivo animal models of atherosclerosis (Figure 1). Mainly two considerations mark the difference between scientific curiosity and clinical need with regard to these technologies.

First, despite considerable progress in cardiovascular medicine, interventional treatment of chronic coronary syndrome or asymptomatic carotid artery stenosis is still a matter of ongoing debate owing to insufficient imaging capabilities for detection of high risk atherosclerotic plaque features. While evidence has become irrefutable that revascularization relying only on angiographical stenosis severity does not lead to reduced mortality in the majority of the cases [53,54], reliable alternatives are lacking. Aiming to overcome this disparity, different approaches identifying prognostically relevant features of plaque morphology and composition derived from intravascular ultrasound [55,56], OCT [57] or NIRS [58], as well as non-invasive imaging modalities, failed to establish sufficient diagnostic accuracy to warrant clinical implementation [59]. In light of the results of previous research, indicating distinguishable intravascular imaging-derived features of high-risk atherosclerotic plaques on the one hand, and the variety of developing molecular imaging technologies that still await prospective evaluation on the other, efforts to push these techniques to clinical translation should continue.

Second, the ability of nanoparticle technologies to specifically target different steps of atherosclerosis pathogenesis also entail the possibility of therapeutic application. Additional incorporation of a therapeutic agent yielding “theranostic” nanoparticles, enables targeted drug delivery as well as diagnostic visualization of the respective target and follow-up on treatment effects. Under ideal conditions, such NPs could provide delivery of high doses of the therapeutic substance to its designated target site with little systemic side effects. Numerous therapeutic and theranostic nanoparticles have been employed in animal models of atherosclerosis with encouraging results. Among those are particles targeting inflammation, neovascularization, lipid-accumulation and macrophages using the same ligands as the diagnostic nanotechnologies described above [7,60]. A similar nanotechnology already under clinical investigation, that just recently gained considerable attention with the presentation of favorable outcomes of the two first phase 3 randomized controlled trials is inclisiran [61,62]. Inclisiran is a small interfering RNA (siRNA), complementary to a portion of the PSCK9-encoding messenger RNA (mRNA). Binding of this siRNA to the RNA-induced silencing complex in hepatocytes causes cleavage of PCSK9-encoding mRNA, sustainably reducing PSCK9-activity and thus LDL-cholesterol levels. The delivery of inclisiran to the liver is achieved by conjugation to N-acetylgalactosamine carbohydrates, known to bind to liver-expressed receptors [63]. However, earlier clinical investigation, establishing the foundation for this mayor innovation in cardiovascular medicine, relied on encapsulation of PCSK9-specific small interfering RNAs into lipid nanoparticles in order to ensure biostability and site-targeted delivery [64,65].

In summary, targeted nanoparticles represent a useful tool for both molecular imaging and therapy of atherosclerotic disease. The combination of deeper understanding of atherosclerosis pathophysiology on a molecular level and improved identification of high-risk atherosclerotic lesions with the increasing evolution of site-targeted therapeutic approaches contains promising synergistic potential.

## References

[1] Naghavi M., Wang H., Lozano R., Davis A., Liang X., Zhou M., Vollset S.E., Abbasoglu Ozgoren A., Abdalla S., Abd-Allah F. (2015). Global, regional, and national age-sex specific all-cause and cause-specific mortality for 240 causes of death, 1990-2013: A systematic analysis for the Global Burden of Disease Study 2013. Lancet.

[2] Libby P. (2002). Inflammation in atherosclerosis. Nature.

[3] Virmani R., Kolodgie F.D., Burke A.P., Farb A., Schwartz S.M. (2000). Lessons From Sudden Coronary Death. Arterioscler. Thromb. Vasc. Biol..

[4] Kanwar S.S., Stone G.W., Singh M., Virmani R., Olin J., Akasaka T., Narula J. (2016). Acute coronary syndromes without coronary plaque rupture. Nat. Rev. Cardiol..

[5] Spratt J.C., Camenzind E. (2004). Plaque stabilisation by systemic and local drug administration. Heart.

[6] Chowdhury M.M., Tawakol A., Jaffer F.A. (2017). Molecular Imaging of Atherosclerosis: A Clinical Focus. Curr. Cardiovasc. Imaging Rep..

[7] Bobo D., Robinson K.J., Islam J., Thurecht K.J., Corrie S.R. (2016). Nanoparticle-Based Medicines: A Review of FDA-Approved Materials and Clinical Trials to Date. Pharm. Res..

[8] Dadfar S.M., Roemhild K., Drude N.I., von Stillfried S., Knüchel R., Kiessling F., Lammers T. (2019). Iron oxide nanoparticles: Diagnostic, therapeutic and theranostic applications. Adv. Drug Deliv. Rev..

[9] Zheng K.H., Schoormans J., Stiekema L.C.A., Calcagno C., Cicha I., Alexiou C., Strijkers G.J., Nederveen A.J., Stroes E.S.G., Coolen B.F. (2019). Plaque Permeability Assessed With DCE-MRI Associates With USPIO Uptake in Patients With Peripheral Artery Disease. JACC Cardiovasc. Imaging.

[10] Figuerola A., Di Corato R., Manna L., Pellegrino T. (2010). From iron oxide nanoparticles towards advanced iron-based inorganic materials designed for biomedical applications. Pharmacol. Res..

[11] Unterweger H., Dézsi L., Matuszak J., Janko C., Poettler M., Jordan J., Bäuerle T., Szebeni J., Fey T., Boccaccini A.R. (2018). Dextran-coated superparamagnetic iron oxide nanoparticles for magnetic resonance imaging: Evaluation of size-dependent imaging properties, storage stability and safety. Int. J. Nanomedicine.

[12] De Sarno F., Ponsiglione A.M., Torino E. (2018). Emerging use of nanoparticles in diagnosis of atherosclerosis disease: A review. AIP Conf. Proc..

[13] Jaffer F.A., Nahrendorf M., Sosnovik D., Kelly K.A., Aikawa E., Weissleder R. (2006). Cellular imaging of inflammation in atherosclerosis using magnetofluorescent nanomaterials. Mol. Imaging.

[14] Nahrendorf M., Jaffer F.A., Kelly K.A., Sosnovik D.E., Aikawa E., Libby P., Weissleder R. (2006). Noninvasive vascular cell adhesion molecule-1 imaging identifies inflammatory activation of cells in atherosclerosis. Circulation.

[15] Bruckman M.A., Jiang K., Simpson E.J., Randolph L.N., Luyt L.G., Yu X., Steinmetz N.F. (2014). Dual-modal magnetic resonance and fluorescence imaging of atherosclerotic plaques in vivo using VCAM-1 targeted tobacco mosaic virus. Nano Lett..

[16] Weissleder R., Nahrendorf M., Pittet M.J. (2014). Imaging macrophages with nanoparticles. Nat. Mater..

[17] Nahrendorf M., Zhang H., Hembrador S., Panizzi P., Sosnovik D.E., Aikawa E., Libby P., Swirski F.K., Weissleder R. (2008). Nanoparticle PET-CT imaging of macrophages in inflammatory atherosclerosis. Circulation.

[18] Tarin C., Carril M., Martin-Ventura J.L., Markuerkiaga I., Padro D., Llamas-Granda P., Moreno J.A., García I., Genicio N., Plaza-Garcia S. (2015). Targeted gold-coated iron oxide nanoparticles for CD163 detection in atherosclerosis by MRI. Sci. Rep..

[19] Guo L., Akahori H., Harari E., Smith S.L., Polavarapu R., Karmali V., Otsuka F., Gannon R.L., Braumann R.E., Dickinson M.H. (2018). CD163+ macrophages promote angiogenesis and vascular permeability accompanied by inflammation in atherosclerosis. J. Clin. Invest..

[20] Cormode D.P., Skajaa T., van Schooneveld M.M., Koole R., Jarzyna P., Lobatto M.E., Calcagno C., Barazza A., Gordon R.E., Zanzonico P. (2008). Nanocrystal Core High-Density Lipoproteins: A Multimodality Contrast Agent Platform. Nano Lett..

[21] Basatemur G.L., Jørgensen H.F., Clarke M.C.H., Bennett M.R., Mallat Z. (2019). Vascular smooth muscle cells in atherosclerosis. Nat. Rev. Cardiol..

[22] Caglayan E., Romeo G.R., Kappert K., Odenthal M., Südkamp M., Body S.C., Shernan S.K., Hackbusch D., Vantler M., Kazlauskas A. (2010). Profilin-1 is expressed in human atherosclerotic plaques and induces atherogenic effects on vascular smooth muscle cells. PLoS One.

[23] Zhang S., Xu W., Gao P., Chen W., Zhou Q. (2020). Construction of dual nanomedicines for the imaging and alleviation of atherosclerosis. Artif. Cells Nanomed. Biotechnol..

[24] Moreno P.R., Purushothaman K.-R., Sirol M., Levy A.P., Fuster V. (2006). Neovascularization in Human Atherosclerosis. Circulation.

[25] Guo L., Harari E., Virmani R., Finn A.V. (2017). Linking hemorrhage, angiogenesis, macrophages, and iron metabolism in atherosclerotic vascular diseases. Arterioscler. Thromb. Vasc. Biol..

[26] Demircioglu F., Hodivala-Dilke K. (2016). αvβ3 Integrin and tumour blood vessels — Learning from the past to shape the future. Curr. Opin. Cell Biol..

[27] Winter P.M., Morawski A.M., Caruthers S.D., Fuhrhop R.W., Zhang H., Williams T.A., Allen J.S., Lacy E.K., Robertson J.D., Lanza G.M. (2003). Molecular Imaging of Angiogenesis in Early-Stage Atherosclerosis With α v β 3 -Integrin-Targeted Nanoparticles. Circulation.

[28] Woodside D.G., Tanifum E.A., Ghaghada K.B., Biediger R.J., Caivano A.R., Starosolski Z.A., Khounlo S., Bhayana S., Abbasi S., Craft J.W. (2018). Magnetic Resonance Imaging of Atherosclerotic Plaque at Clinically Relevant Field Strengths (1T) by Targeting the Integrin α4β1. Sci. Rep..

[29] Yahagi K., Kolodgie F.D., Otsuka F., Finn A.V., Davis H.R., Joner M., Virmani R. (2016). Pathophysiology of native coronary, vein graft, and in-stent atherosclerosis. Nat. Rev. Cardiol..

[30] Ramirez-Carracedo R., Tesoro L., Hernandez I., Diez-Mata J., Filice M., Toro R., Rodriguez-Piñero M., Zamorano J.L., Saura M., Zaragoza C. (2018). Non-invasive detection of extracellular matrix metalloproteinase inducer EMMPRIN, a new therapeutic target against atherosclerosis, inhibited by endothelial nitric oxide. Int. J. Mol. Sci..

[31] Pirillo A., Norata G.D., Catapano A.L. (2013). LOX-1, OxLDL, and atherosclerosis. Mediators Inflamm..

[32] Li D., Patel A.R., Klibanov A.L., Kramer C.M., Ruiz M., Kang B.Y., Mehta J.L., Beller G.A., Glover D.K., Meyer C.H. (2010). Molecular imaging of atherosclerotic plaques targeted to oxidized LDL receptor LOX-1 by SPECT/CT and magnetic resonance. Circ. Cardiovasc. Imaging.

[33] Wen S., Liu D.F., Liu Z., Harris S., Yao Y.Y., Ding Q., Nie F., Lu T., Chen H.J., An Y.L. (2012). OxLDL-targeted iron oxide nanoparticles for in vivo MRI detection of perivascular carotid collar induced atherosclerotic lesions in ApoE-deficient mice. J. Lipid Res..

[34] Pellico J., Fernández-Barahona I., Benito M., Gaitán-Simón Á., Gutiérrez L., Ruiz-Cabello J., Herranz F. (2019). Unambiguous detection of atherosclerosis using bioorthogonal nanomaterials. Nanomed. Nanotechnol. Biol. Med..

[35] Otsuka F., Yasuda S., Noguchi T., Ishibashi-ueda H. (2016). Pathology of coronary atherosclerosis and thrombosis. Cardiovasc. Diagn. Ther..

[36] van Tilborg G.A.F., Vucic E., Strijkers G.J., Cormode D.P., Mani V., Skajaa T., Reutelingsperger C.P.M., Fayad Z.A., Mulder W.J.M., Nicolay K. (2010). Annexin A5-Functionalized Bimodal Nanoparticles for MRI and Fluorescence Imaging of Atherosclerotic Plaques. Bioconjug. Chem..

[37] A Zayed M., R Abendschein D., Vemuri C., Lu D., Detering L., Liu Y., K Woodard P. (2016). Natriuretic Peptide Receptor-C is Up-Regulated in the Intima of Advanced Carotid Artery Atherosclerosis. J. Med. Surg. Pathol..

[38] Liu Y., Luehmann H.P., Detering L., Pressly E.D., McGrath A.J., Sultan D., Nguyen A., Grathwohl S., Shokeen M., Zayed M. (2019). Assessment of Targeted Nanoparticle Assemblies for Atherosclerosis Imaging with Positron Emission Tomography and Potential for Clinical Translation. ACS Appl. Mater. Interfaces.

[39] Kwon S., Jeon S., Lee S., Yeol H., Hee J., Choi D., Kim J., Kim J., Hyung J., Kim D. (2018). Biomaterials Thrombin-activatable fl uorescent peptide incorporated gold nanoparticles for dual optical / computed tomography thrombus imaging. Biomaterials.

[40] Zhong Y. (2019). Polydopamine-modi fi ed dual-ligand nanoparticles as highly effective and targeted magnetic resonance / photoacoustic dual-modality thrombus imaging agents. Int. J. Nanomed..

[41] Partida R.A., Libby P., Crea F., Jang I.K. (2018). Plaque erosion: A new in vivo diagnosis and a potential major shift in the management of patients with acute coronary syndromes. Eur. Heart J..

[42] Michail M., Serruys P.W., Stettler R., Crake T., Torii R., Tenekecioglu E., Zeng Y., Onuma Y., Mathur A., Bourantas C.V. (2017). Intravascular multimodality imaging: Feasibility and role in the evaluation of coronary plaque pathology. Eur. Heart J. Cardiovasc. Imaging.

[43] Bozhko D., Osborn E.A., Rosenthal A., Verjans J.W., Hara T., Kellnberger S., Wissmeyer G., Ovsepian S.V., McCarthy J.R., Mauskapf A. (2017). Quantitative intravascular biological fluorescence-ultrasound imaging of coronary and peripheral arteries in vivo. Eur. Heart J. Cardiovasc. Imaging.

[44] Hara T., Ughi G.J., McCarthy J.R., Erdem S.S., Mauskapf A., Lyon S.C., Fard A.M., Edelman E.R., Tearney G.J., Jaffer F.A. (2017). Intravascular fibrin molecular imaging improves the detection of unhealed stents assessed by optical coherence tomography in vivo. Eur. Heart J..

[45] Jaffer F.A., Calfon M.A., Rosenthal A., Mallas G., Razansky R.N., Mauskapf A., Weissleder R., Libby P., Ntziachristos V. (2011). Two-dimensional intravascular near-infrared fluorescence molecular imaging of inflammation in atherosclerosis and stent-induced vascular injury. J. Am. Coll. Cardiol..

[46] Vinegoni C., Botnaru I., Aikawa E., Calfon M.A., Iwamoto Y., Folco E.J., Ntziachristos V., Weissleder R., Libby P., Jaffer F.A. (2011). Indocyanine green enables near-infrared fluorescence imaging of lipid-rich, inflamed atherosclerotic plaques. Sci. Transl. Med..

[47] Stein-Merlob A.F., Hara T., McCarthy J.R., Mauskapf A., Hamilton J.A., Ntziachristos V., Libby P., Jaffer F.A. (2017). Atheroma Susceptible to Thrombosis Exhibit Impaired Endothelial Permeability In Vivo as Assessed by Nanoparticle-Based Fluorescence Molecular Imaging. Circ. Cardiovasc. Imaging.

[48] Galkina E., Ley K. (2007). Vascular adhesion molecules in atherosclerosis. Arterioscler. Thromb. Vasc. Biol..

[49] Kee P.H., Kim H., Huang S., Laing S.T., Moody M.R., Vela D., Klegerman M.E., McPherson D.D. (2014). Nitric Oxide Pretreatment Enhances Atheroma Component Highlighting in Vivo with Intercellular Adhesion Molecule-1-Targeted Echogenic Liposomes. Ultrasound Med. Biol..

[50] Bourantas C.V., Jaffer F.A., Gijsen F.J., Van Soest G., Madden S.P., Courtney B.K., Fard A.M., Tenekecioglu E., Zeng Y., Van Der Steen A.F.W. (2017). Hybrid intravascular imaging: Recent advances, technical considerations, and current applications in the study of plaque pathophysiology. Eur. Heart J..

[51] Wang B., Karpiouk A., Yeager D., Amirian J., Litovsky S., Smalling R., Emelianov S. (2012). In vivo Intravascular Ultrasound-guided Photoacoustic Imaging of Lipid in Plaques Using an Animal Model of Atherosclerosis. Ultrasound Med. Biol..

[52] Wang B., Yantsen E., Larson T., Karpiouk A.B., Sethuraman S., Su J.L., Sokolov K., Emelianov S.Y. (2009). Plasmonic intravascular photoacoustic imaging for detection of macrophages in atherosclerotic plaques. Nano Lett..

[53] Al-Lamee R., Thompson D., Dehbi H.M., Sen S., Tang K., Davies J., Keeble T., Mielewczik M., Kaprielian R., Malik I.S. (2017). Percutaneous coronary intervention in stable angina (ORBITA): A double-blind, randomised controlled trial. Lancet.

[54] Hochman J.S. International Study of Comparative Health Effectiveness With Medical and Invasive Approaches: Primary report of clinical outcomes. Proceedings of the AHA 2019.

[55] Stone P.H., Saito S., Takahashi S., Makita Y., Nakamura S., Kawasaki T., Takahashi A., Katsuki T., Nakamura S., Namiki A. (2012). Prediction of progression of coronary artery disease and clinical outcomes using vascular profiling of endothelial shear stress and arterial plaque characteristics: The PREDICTION study. Circulation.

[56] Stone G.W., Maehara A., Lansky A.J., De Bruyne B., Cristea E., Mintz G.S., Mehran R., McPherson J., Farhat N., Marso S.P. (2011). A prospective natural-history study of coronary atherosclerosis. N. Engl. J. Med..

[57] Prati F., Romagnoli E., Gatto L., La Manna A., Burzotta F., Ozaki Y., Marco V., Boi A., Fineschi M., Fabbiocchi F. (2019). Relationship between coronary plaque morphology of the left anterior descending artery and 12 months clinical outcome: The CLIMA study. Eur. Heart J..

[58] Oemrawsingh R.M., Cheng J.M., García-García H.M., Van Geuns R.J., De Boer S.P.M., Simsek C., Kardys I., Lenzen M.J., Van Domburg R.T., Regar E. (2014). Near-infrared spectroscopy predicts cardiovascular outcome in patients with coronary artery disease. J. Am. Coll. Cardiol..

[59] Bourantas C.V., Garcia-Garcia H.M., Torii R., Zhang Y.J., Westwood M., Crake T., Serruys P.W. (2016). Vulnerable plaque detection: An unrealistic quest or a feasible objective with a clinical value?. Heart.

[60] Flores A.M., Ye J., Jarr K.-U., Hosseini-Nassab N., Smith B.R., Leeper N.J. (2019). Nanoparticle Therapy for Vascular Diseases. Arterioscler. Thromb. Vasc. Biol..

[61] Scott Wright R., Rochester M., Mayo Clinic ORION-10 Inclisiran for subjects with ACSVD and elevated low-density lipoprotein cholesterol. Proceedings of the AHA 2019.

[62] Ray K.K. Impact of inclisiran on LDL-C over 18 months in patients with ASCVD or risk-equivalent - Results of the Phase 3 ORION-11 trial. Proceedings of the Late Breaking Science in Prevention 1 Session at ESC Congress 2019.

[63] Levin A.A. (2019). Treating disease at the RNA level with oligonucleotides. N. Engl. J. Med..

[64] Fitzgerald K., White S., Borodovsky A., Bettencourt B.R., Strahs A., Clausen V., Wijngaard P., Horton J.D., Taubel J., Brooks A. (2017). A Highly Durable RNAi Therapeutic Inhibitor of PCSK9. N. Engl. J. Med..

[65] Fitzgerald K., Frank-Kamenetsky M., Shulga-Morskaya S., Liebow A., Bettencourt B.R., Sutherland J.E., Hutabarat R.M., Clausen V.A., Karsten V., Cehelsky J. (2014). Effect of an RNA interference drug on the synthesis of proprotein convertase subtilisin/kexin type 9 (PCSK9) and the concentration of serum LDL cholesterol in healthy volunteers: a randomised, single-blind, placebo-controlled, phase 1 trial. Lancet.

